# Evaluate how steaming and sulfur fumigation change the microstructure, physicochemical properties and *in vitro* digestibility of *Gastrodia elata* Bl. starch

**DOI:** 10.3389/fnut.2022.1087453

**Published:** 2023-01-05

**Authors:** Jinjie Guan, Zhuowen Chen, Lanping Guo, Xiuming Cui, Tingting Xu, Fen Wan, Tao Zhou, Chengxiao Wang, Ye Yang

**Affiliations:** ^1^Faculty of Life Science and Technology, Kunming University of Science and Technology, Kunming, Yunnan, China; ^2^Yunnan Provincial Key Laboratory of Panax Notoginseng, Kunming, China; ^3^China Academy of Chinese Medical Sciences, Beijing, China; ^4^Resource Institute for Chinese and Ethnic Materia Medica, Guizhou University of Traditional Chinese Medicine, Guiyang, China

**Keywords:** *Gastrodia elata* starch, steaming, sulfur fumigation, edible quality, sulfur dioxide gas

## Abstract

The sulfur dioxide gas (SO_2_) generated by sulfur burning can improve the appearance quality of food and enhance the storage time. However, excessive sulfur dioxide will pollute the environment and cause deterioration of food quality, and even the high residual levels can increase the risk of cancer. As *Gastrodia elata* Blume is prone to corruption during processing, sulfur fumigation is often used for preservation. In this study, spectral analysis and Texture Profile Analysis (TPA) were used to investigate the effects of traditional sulfur fumigation processing on the morphology quality, edible quality and structural characteristics of *G. elata*. The results showed that compared with direct drying, the pH decreased by 0.399 of the sulfur fumigated after steamed treatment *G. elata*, and the morphology quality, pasting ability and gel edible quality of the starch were significantly improved. In addition, it was suggested that sulfur fumigation after steaming could promote the release of molecular chains from starch granules and thus enhance the cross-linking between molecules, which explained the reason for the improve of starch edible quality. This study can provide technical and theoretical support for improving the quality of starch rich foods, replacing sulfur fumigation and reducing potential environmental hazards.

## 1. Introduction

Sulfur fumigation is often used for food mildew prevention and storage. However, SO_2_ and its derivatives generated in the process of sulfur fumigation are the main pollutants in the atmosphere that are harmful to the environment (1). At the same time, excessive SO_2_ residues in food have carcinogenic effects on human health. Therefore, to maintain the environment and consumer health, the residual amount of SO_2_ in food processing is limited strictly (1). The Chinese government stipulates that the SO_2_ content in food shall not exceed 100 mg/kg (2), and the sulfur-fumigated Chinese herbal medicine should be less than 400 mg/kg (3).

*Gastrodia elata* Blume (*G. elata*) is the rhizome of the genus *Gastrodia* belonging to family *Orchidaceae*. It has the function of relieving wind and spasm, calming liver Yang, dispelling wind and dredging collaterals. It is traditionally used as treatment for manage convulsions in children, epilepsy, tetanus, headache and dizziness, hand and foot failure, numbness of limbs, rheumatism, and arthralgia (4). *G. elata* is a kind of traditional medicine and food widely used in many Asian countries (5). In 2002, *G. elata* was included in the list of items potentially used as health food by the Chinese government, representing a “health food” in the legal sense. In 2019, *G. elata* was designated by the Chinese Health Commission as a pilot variety for the management of “substances that are both food and Chinese herbal medicines according to tradition” and represented a “medicine-food homology” in the legal sense. With *G. elata* becoming a food of legal significance, its edible range and usage are also expanding rapidly.

The common drying processes of *G. elata* are sun drying, hot air drying and vacuum freeze drying. Sun drying efficiency is low, but the drying cost is also low. Vacuum freeze-drying is the best method to maintain the appearance and active ingredients, but it requires expensive equipment and high drying costs. However, due to the conditions of the production base and the processing cost, hot air drying is the most commonly used processing technology. *G. elata* is rich in starch and sugar, with levels as high as 12.5 and 8%, respectively. Nonetheless, the water content of fresh *G. elata* is as high as 75%, with an average fresh weight of more than 100 g, which decreases the rate of water loss during the drying process and increases bacterial and fungal contamination and rotting because of the high levels of sugars and starch (6).

Sulfur fumigation can prevent food decay caused by bacterial and fungal pollution and can improve the appearance quality of food, such as copra and yam (7). In addition, the presteaming treatment can avoid the composition change of fresh food due to its own metabolic reaction. However, excessive sulfur fumigation will cause sulfur dioxide residue, which is harmful to human health. The sulfur fumigation process is a traditional Chinese method for processing *G. elata*, which can prevent enzymatic browning caused by the decomposition of gastrodin and phenolic substances by enzymes during the processing of *G. elata*, and the SO_2_ produced by sulfur combustion can increase the color while preventing mildew (4).

High-quality *G. elata* is characterized by strong waxiness and a high degree of sweetness. The hardness and viscoelasticity of foods with high starch content are mainly affected by starch composition and structure. For example, the amylose content and the molecular chain size are positively correlated with the hardness of rice, while the content of amylopectin and short-chain starch is positively correlated with the viscosity of rice (8). Long amylopectin chains (DP > 35) were positively correlated with the degree of swelling (9). In addition, the cooking characteristics of food are also related to the composition and structure of starch, such as the higher disintegration temperature of starch crystals, which prolongs the cooking time of rice (10). Therefore, dry heat treatment, acetic acid acetylation and hydrochloric acid hydrolysis are used to modify starch to increase its edible quality (11–13). Jyothi et al. (14) soaked cassava starch with 2% sodium metabisulfite and found that the starch viscosity first increased and then decreased with the extension of soaking time, which increased the storage time and solubility of cassava starch. Sodium metabisulfite will dissociate into sulfurous acid in aqueous solution, and the SO_2_ from sulfur combustion products combines with water to generate sulfurous acid, thus changing the structure of starch (15).

To preserve the nutritional ingredients in food, fresh food is usually presteamed and then dried for preservation. However, this high temperature condition will change the composition and structural characteristics of starch. For example, the amylose content of corn and potato increases after steaming, and the crystal structure of lily starch changes from type B to type A after heat treatment (16, 17). The previous research of this study shows that the taste of *G. elata* fumigated with sulfur in the traditional way was significantly increased in terms of chewiness, but the mechanism is still unclear. Zhuowen Chen et al. (15) have shown that the proportion of amylose in yam after sulfur fumigation is higher than that without sulfur fumigation. The starch content of *G. elata* is similar to that of yam and cassava, accounting for approximately 50% of its dry weight. We infer that this may be because sulfur fumigation leads to the breaking of amylopectin and the formation of short amylose, which increases the cross-linking degree between starch molecules, increases the hardness of starch gel, and thus increases the chewiness of *G. elata*. However, the inference and the mechanism of action need to be further studied.

To explain the scientific connotation of the influence of traditional processing technology of sulfur fumigation after steaming on the color, viscosity and chewiness quality of *G. elata* starch, this study explored the influence of sulfur fumigation after steaming on the crystallinity and viscoelasticity of starch during processing and revealed the relationship between the edible quality of starch and the change in starch structure. This study can provide data support for the scientific nature of traditional processing technology of *G. elata* and provide a theoretical basis for the rational use of sulfur fumigation in food and the control of potential environmental hazards.

## 2. Materials and methods

### 2.1. Materials

Fresh *G. elata Bl. f. glauca S. Chow* (Grade II, 2 years old, weighing 100–200 g per *G. elata*, with 75% water content) was collected in December 2021 from Xiaocaoba Town, Zhaotong, Yunnan, China (104° 15′E, 27° 46′ N), for use in this study. Sulfur was purchased from Sinopharm Chemical Reagent Co., Ltd., (Shanghai, China, No. 80120528). The steaming equipment is an electric cooker (ZG500, Medical Equipment Factory of Boxun Industrial Co., Ltd., Shanghai, China). All chemical reagents used in this study were of analytical grade.

### 2.2. Steaming and sulfur fumigation of *G. elata*

After the fresh *G. elata* was cleaned, it was divided into steam treatment (St) and nonsteam treatment (NSt). In steam treatment, *G. elata* is steamed in a steamer for 30 min. After drying the surface moisture, *G. elata* was removed for sulfur fumigation treatment (Sf) and nonsulfur fumigation treatment (NSf) from the St and NSt treatments. The Sf treatment according to the ratio of *G. elata*:sulfur (20:1) and placed in a fumigation box (60 cm × 45 cm × 35 cm). After the sulfur was ignited, the fumigation box was capped, and a small hole of approximately 1 cm^2^ was left for ventilation. After fumigation for 90 min, it was removed. All *G. elata* were placed into the electric constant temperature blast drying oven (DGG-9123AD, Senxin Experimental Instrument Co., Ltd., Shanghai, China) with 50°C hot air and dried for 5 days, and the final water content was approximately 5%. After drying, the *G. elata* was powdered and passed through the 200 mesh sieve. The starch was extracted following the method of Jiang et al. (18).

### 2.3. Determination of the pH and sulfur dioxide residue analysis of *G. elata* powder

The pH of *G. elata* powder was determined following the method of Liu et al. (19) with a slight modification. Take 0.8 g of *G. elata* powder, add 20 mL of distilled water for cold immersion for 12 h, then extract it by ultrasonic for 30 min, and measure it with a pH meter (STARTER-3100, Aohaosi Instrument Co., Ltd., Shanghai, China). The sulfur dioxide residue was determined following the method of Kang et al. (4).

### 2.4. Determination of chromaticity, solubility, swelling power, amylose content, and paste transparency of *G. elata* starch

The chromaticity is measured with a colorimeter (SC-10, Sanenchi Technology Co., Ltd., Shenzhen, China). Solubility and swelling power were determined following the method of Lu et al. (20) with a slight modification. Take 0.5 g sample powder, add 25 mL distilled water, mix well, shake and paste it in boiling water bath for 30 min, then cool it and centrifuge it at 4,000 r/min for 25 min, take the supernatant, dry it to constant weight and weigh it.


(1) Solubility(%)=(M1/M2)×100%

(2) Swellingpower(%)=M3/[M2×(1-S)]

(3) $$\text{Whiteness}=100-\sqrt{{\left(100-L\right)}^{2}+a^{2}+b^{2}}$$

where *M1* is the starch mass in the supernatant after drying, *M2* is the sample mass, *M3* is the sediment mass after centrifugation, *S* is the sample solubility.

Amylose content following the method of Uarrota et al. (21) with a slight modification. Take 50 mg of *G. elata* starch, mix it with 5 mL of 0.5 mol/L KOH solution, dilute it 10-fold, take 1 mL of solution, adjust the pH to 3.5 with 0.1 mol/L HCl, add 0.1 mL iodine reagent (20 mg KI–2 mg I/mL, AR), and measure the absorbance at 625 nm. Calculate the content according to the following standard curve:


Y=1.754X-0.4327,R=20.999.

Use the mixed solution with amylose content (0, 20, 40, 60, and 80%) to measure the absorbance at 625 nm to obtain the standard curve. Where *Y* is the amylose content (%) in the mixed standard solution, and *X* is the absorbance measured at 625 nm.

Paste transparency was determined following the method of Correia et al. (22). *G. elata* starch was stirred in a boiling water bath for 1 h to prepare a 2% starch suspension. After cooling at 25°C, the transmittance was measured at 620 nm.

### 2.5. Texture characteristics of *G. elata* starch gel (TPA)

Texture characteristics were determined following the method of Lu et al. (20) with a slight modification. Mix *G. elata* starch and water to prepare a 10% aqueous starch solution, shake and mix in a boiling water bath for 30 min. Then, the sample was refrigerated for 12 h at 4°C. After the formation of the gel, it was removed and restored to room temperature. Texture analyzer (TA. XT plus, London, UK) was used for measurement, and P/0.5 was used as the probe. The parameters were set as follows: the test speed was 1 mm/s, the test distance was 13 mm, and the trigger force was 2 g. Hardness, adhesiveness, springiness, cohesiveness, gumminess, chewiness, and resilience were recorded.

### 2.6. Fourier transform infrared spectroscopy

Following the method of Lu et al. (20), the FT-IR spectra of the samples were determined using FT-IR spectroscopy (Sensor 27, Bruker Inc., Saarbrucken, Germany). Mix and grind *G. elata* starch and dried KBr and scan them in the wavenumber range of 500–4,000 cm^–1^ at room temperature after tablet pressing.

### 2.7. X-ray diffraction

Following the method of Lu et al. (20) with a slight modification to determine the crystalline structure of starch by XRD (D8 Advance, Bruker Inc., Saarbrucken, Germany). The parameters were set as follows: the diffraction angle was 2 θ, with a range of 4°∼70°, and the scanning rate was 4°/min. The relative crystallinity was calculated using JADE 6.5 (Java Agent Development Framework) software.

### 2.8. Measurement of paste properties with a rapid viscosity analyzer

Following the method of Li et al. (17) with a slight modification to determine the adhesive properties of *G. elata* starch by rapid viscosity analyzer (RVA-Taskmaster, Perton Inc., Stockholm, Sweden). Dissolve 1.25 g of *G. elata* starch in 25 mL of water, stir for 30 min, mix well, and measure it according to the method of rice configuration file in RVA.

### 2.9. Thermogravimetric analysis

Following the method of Ma et al. (23), the thermogravimetric properties of *G. elata* starch were determined by STA (STA-2500, NETZSCH Inc., Selb, Germany). The test temperature was 30°C–600°C, nitrogen was the ambient gas, and the rising temperature was 10°C/min.

### 2.10. Rheological measurement

The rheological properties of the samples were determined using a rheometer (DHR-2, TA Instruments Inc., New Castle, DE, USA). The test was conducted at 25°C using 40 mm parallel plates. Take 0.25 g of starch to prepare a 5% starch suspension (mix it evenly in boiling water for 10 min and cool it to room temperature), then transfer it to a rheological plate and balance it for 3 min. According to the method of Lu et al. (20), frequency scanning was carried out at 1% strain in the angular frequency range of 0.1–100 rad/s. The shear viscosity was measured between 0.1 and 100 s^–1^. Data were fitted by the power law equation:


$$\sigma =\text{K}\left(\gamma \right){}^{n}$$

where σ is the shear stress (Pa), K is the consistency coefficient (Pa⋅s^–1^), γ is the shear rate (s^–1^), and n is the non-Newtonian exponent.

### 2.11. Molecular weight analysis with high-performance gel permeation chromatography

Accurately weigh the starch sample and dextran standards (Sigma-Aldrich Co., Ltd., Darmstadt, Germany) to prepare 5 mg/mL in 0.2 M NaOH solution, hydrolyzed at 120°C for 1 h, centrifuged at 12,000 rpm for 10 min, and a 0.22 μm microporous filter membrane was used, and then the sample was transferred into a 1.8 mL injection vial. 0.05 M NaCl solution was used as the mobile phase for analysis at a flow rate of 0.6 mL/min. BRT105-104-102 series gel column (8 × 300 mm), column temperature: 40°C, injection volume: 20 μL, detector: differential detector RI-10A. A standard curve was drawn with the retention time (RT) of dextran standards on the *X*-axis and lgMw and lgMn on the *Y*-axis.

lgMw-RT of the correction curve equation:


(1) Y=-0.1932X+12.211R=20.9929

lgMn-RT of the correction curve equation:


(2) Y=-0.1792X+11.494R=20.9918

In (1), *Y* is lgMw, and *X* is the retention time (RT).

In (2), *Y* is lgMn, and *X* is the retention time (RT).

### 2.12. *In vitro* digestion characteristics of starch

Following the method of Zeng et al. (24). Add 7.5 mL of acetic acid buffer (0.2 mol/L, pH 5.2) to 100 mg of sample, keep stirring in boiling water bath, cool to room temperature, add 5 mL of enzyme solution (each mL contains 294 U of porcine pancreatic amylase and 15 U of glucoamylase), shake and mix at 37°C for enzymatic hydrolysis, take 0.5 mL of sample every 20 min, and immediately add 4 mL of absolute ethanol to terminate the reaction. Monitor continuously for 4 h. The glucose content of the sample to be tested was determined by the Ghose method. Different starch contents were calculated according to the following formula: rapidly digesting starch (RDS, digestion within 20 min), slowly digesting starch (SDS, digestion within 20–120 min), and resistant starch (RS, residual starch after digestion for 120 min).


(1) RDS(%)=[(G-20FG)×0.9]/(TS×100)

(2) SDS(%)=[(G-120G)20×0.9]/(TS×100)

(3) RS(%)=(TS-RDS-SDS)/(TS×100)

(4) Y=3.7857X-0.4174,R=20.998

G_20_ is the glucose content in the solution at 20 min of enzymatic hydrolysis, G_120_ is the glucose content in the solution at 120 min of enzymatic hydrolysis, FG is the free sugar content in the starch solution at 0 min, TS is the total mass of the starch sample used, *Y* is the glucose content (mg), and *X* is the absorbance measured at 540 nm.

### 2.13. Scanning electron microscopy

The microstructure of the starch powder was observed with SEM (S-3400 N, Hitachi Inc., Tokyo, Japan), and the sample powder was fixed on the sample rack and placed in the instrument for observation.

### 2.14. Atomic force microscopy

AFM (5500LS, Agilent Inc., Palo Alto, CA, USA) was used to observe the microscopic morphology of the starch paste. One hundred milligrams of the sample was added to 20 mL of distilled water, stirred and mixed in a boiling water bath, and the paste was continued for 10 min and diluted with hot water to 50 μg/mL. The sample was placed on the mica surface and observed after drying.

### 2.15. Confocal laser scanning microscopy

Following the method of Ma et al. with a slight modification (23). Samples of 0.5 g starch mixed with 9.5 mL deionized water were stirred for 20 min. Then, they were stirred and mixed in a boiling water bath for 10 min. Wet starch samples (30 μL) were then stained by fluorescein isothiocyanate (FITC, 0.2 mg/mL, 30 μL). Images were collected using CLSM (TI-E-A1R, NIKON Inc., Tokyo, Japan) with an excitation wavelength of 488 nm and an emission wavelength of 450–540 nm.

### 2.16. Statistical analysis

All experiments were repeated at least in triplicate. Data were analyzed using SPSS 26.0 (SPSS Inc., Chicago, IL, USA) for statistical significance (*P* < 0.05) under Tukey’s test. All values are expressed as the mean ± standard deviation. Drawing was performed with Prism 9.0 (GraphPad Inc., San Diego, CA, USA).

## 3. Results

### 3.1. Effect of steaming and sulfur fumigation on the sulfur dioxide residue content and pH of *G. elata*

**FIGURE 1:**
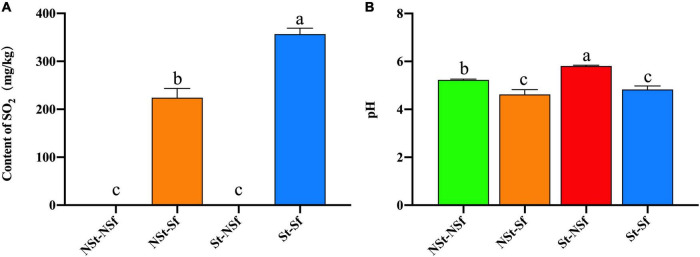
Residual sulfur dioxide content after processing of *Gastrodia elata* **(A)**, Effect of steaming and sulfur fumigation on the pH of *G. elata* powder **(B)**. NSt-NSf, nonsteam and nonsulfur fumigation treatment; NSt-Sf, nonsteam and sulfur fumigation treatment; St-NSf, steam and nonsulfur fumigation treatment; St-Sf, steam and sulfur fumigation treatment.

No residual sulfur dioxide was detected in the *G. elata* with NSt-NSf and St-NSf treatment, but the residual sulfur dioxide in the *G. elata* with sulfur fumigation treatment (NSt-Sf and St-Sf) was 223.67 and 356.67 mg/kg, respectively, which indicate that the samples had been successfully pretreated by sulfur fumigation. The sulfur dioxide residue of *G. elata* with St Sf treatment was 1.59-fold that with NSt-Sf treatment, which indicates that the steamed *G. elata* more easily absorbed sulfur dioxide than the nonsteamed *G. elata* (Figure 1A).

The *G. elata* powder pH of the St-NSf and St-Sf treatments was 0.58 and 0.21 higher than that of the NSt-NSf and NSt-Sf treatments, respectively. The SO_2_ produced by sulfur fumigation dissolved in water and formed H_2_SO_3_, resulting in the pH of *G. elata* reduced. NSt-Sf and St-Sf treatments reduced the pH of *G. elata* powder lower than NSt-NSf and St-NSf treatment by 0.61 and 0.98, respectively. However, sulfur fumigation had no significant effect on the pH of *G. elata* regardless of steaming treatment (Figure 1B).

### 3.2. Effects of steaming and sulfur fumigation on paste transparency and amylose content of *G. elata* starch

**FIGURE 2:**
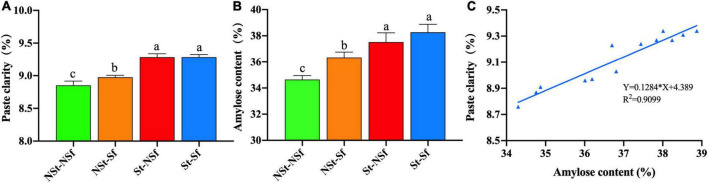
Effects of steaming and sulfur fumigation on paste transparency **(A)**, amylose content **(B)**, and the correlation between paste transparency and amylose content **(C)** of *Gastrodia elata* starch.

After St treatment, the transparency of *G. elata* starch paste was significantly higher than that of NSt. Compared with the NSt-NSf and NSt-Sf treatments, the paste transparency after the St-NSf and St-Sf treatments increased by 1.05 and 1.03-fold, respectively. When fresh *G. elata* was directly sulfur fumigation treated without steaming, the paste transparency of starch was also significantly increased. The effect of the NSt-Sf treatment was 1.01-fold higher than that of the NSt-NSf treatment. Sulfur fumigation had no significant effect on the starch paste transparency of steamed *G. elata*, and there was no significant difference in the starch paste transparency between the St-NSf and St-Sf treatments (Figure 2A).

After St treatment, the amylose content of *G. elata* was significantly higher than that of NSt treatment. Compared with NSt-NSf and NSt-Sf treatments, the amylose content after St-NSf and St-Sf treatments was increased by 1.08 and 1.05-fold, respectively. The amylose content of *G. elata* subjected to NSt-Sf and St-Sf treatment was 1.05 and 1.02-fold higher than that of NSt-NSf and St-NSf treatment without sulfur fumigation, respectively. In the NSt treatment, Sf treatment significantly increased the amylose content. After St treatment, Sf treatment had no significant effect on the amylose content of *G. elata* (Figure 2B). The paste transparency of *G. elata* starch increased with increasing amylose content, resulting in a very significant positive correlation (Figure 2C, *P* < 0.0001).

### 3.3. Effects of steaming and sulfur fumigation treatment on the color of *G. elata* and starch

**FIGURE 3:**
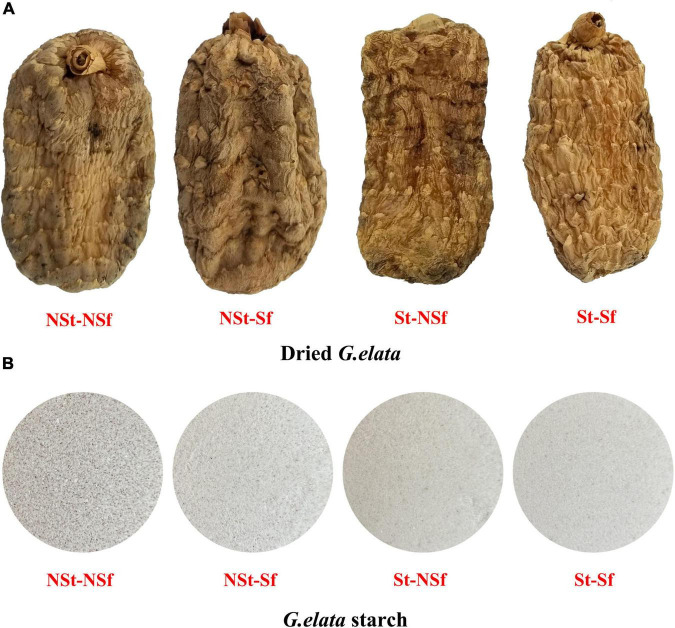
Effects of steaming and sulfur fumigation treatment on the morphology of *Gastrodia elata* and starch **(A,B)**.

After St treatment of fresh *G. elata*, the whiteness of starch was significantly increased compared with that of NSt treatment (Figure 3B). The starch whiteness of St-NSf and St-Sf were significantly increased by 1.10 and 1.03-fold, respectively, compared with NSt-NSf and NSt-Sf. Sf treatment also significantly increased the whiteness of *G. elata* starch. Compared with the NSt-NSf and St-NSf treatments, the whiteness of *G. elata* starch after the NSt-NSf and St-NSf treatments increased by 1.11 and 1.04-fold, respectively (Figure 3, Table 1).

**TABLE 1:** Effect of steaming and sulfur fumigation treatment on the whiteness of *Gastrodia elata* starch.

Sample	*L*	*a*	*b*	Whiteness
NSt-NSf	54.73 ± 0.06d	6.07 ± 0.12a	15.70 ± 0.10b	51.71 ± 0.09c
NSt-Sf	60.77 ± 0.23b	4.00 ± 0.44b	16.17 ± 0.32a	57.38 ± 0.33b
St-NSf	60.40 ± 0.17c	4.03 ± 0.15b	16.23 ± 0.15a	57.01 ± 0.22b
St-Sf	62.40 ± 0.20a	4.50 ± 0.30b	15.10 ± 0.27c	59.23 ± 0.20a

The values are expressed as the mean ± standard deviation of triplicate experiments. Values in the same column with different letters indicate significant differences (*P* < 0.05).

NSt-NSf, nonsteam and nonsulfur fumigation treatment; NSt-Sf, nonsteam and sulfur fumigation treatment; St-NSf, steam and nonsulfur fumigation treatment; St-Sf, steam and sulfur fumigation treatment; *L*, Luminosity; *a*, the range of red to green; *b*, the range of yellow to blue.

### 3.4. Effect of steaming and sulfur fumigation on the solubility and swelling power of *G. elata* starch

**FIGURE 4:**
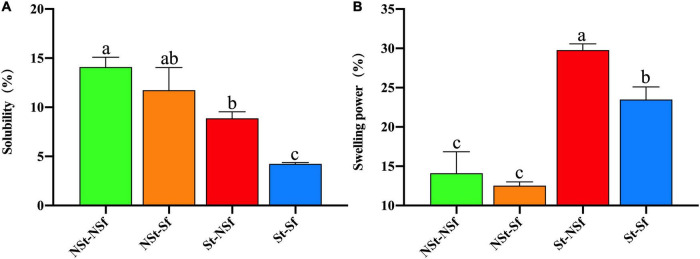
Effect of steaming and sulfur fumigation on solubility **(A)** and swelling power **(B)** of *Gastrodia elata* starch.

After St treatment, the starch solubility of *G. elata* was significantly lower than that of NSt treatment starch. Compared with the NSt-NSf and NSt-Sf treatments, the solubility of starch after the St-NSf and St-Sf treatments were decreased to 63 and 36%, respectively. The Sf treatment also reduced the solubility of starch compared with the NSt-NSf and St-NSf treatments, and the solubility of starch after the NSt-Sf and St-Sf treatments decreased to 83 and 48%, respectively (Figure 4A). The St treatment significantly increased the starch swelling power, and compared with the NSt-NSf and NSt-Sf treatments, the swelling power of starch after the St-NSf and St-Sf treatments was significantly increased by 211 and 187%, respectively. Sf treatment had no significant effect on the swelling of NSt-treated *G. elata* starch. No significant difference was found between the NSt-NSf and NSt-Sf treatments. The St-Sf treatment significantly reduced the swelling of *G. elata* starch compared with the St-NSf treatment. Compared with St-NSf-treated starch, the swelling of St-Sf-treated starch was decreased to 79% (Figure 4B).

### 3.5. Effect of steaming and sulfur fumigation on the TPA of *G. elata* starch gel

The St treatment of fresh *G. elata* led to a significant increase in the hardness and adhesiveness of starch gel. The hardness of St-NSf and St-Sf was 1.18 and 1.78-fold higher than that of NSt-NSf and NSt-Sf, respectively. The adhesiveness of St-NSf and St-Sf was 3.48 and 2.91-fold higher than that of NSt-NSf and NSt-Sf, respectively. St treatment decreased the cohesiveness and resilience by increasing the viscosity of the *G. elata* starch gel. The cohesiveness of the St-NSf and St-Sf treatments was 79 and 85% lower than that of the NSt-NSf and NSt-Sf treatments, and the resilience of the St-NSf and St-Sf treatments was 83 and 62% lower than that of the NSt-NSf and NSt-Sf treatments, respectively. The St treatment had no significant effect on the springiness, gumminess and chewiness of *G. elata* starch gel (Table 2).

**TABLE 2:** Texture characteristics of starch gel.

Sample	Hardness (N)	Adhesiveness (g/sec)	Springiness	Cohesiveness	Gumminess	Chewiness	Resilience
NSt-NSf	13.10 ± 0.60c	19.20 ± 1.42d	0.93 ± 0.00a	0.86 ± 0.00a	11.24 ± 0.49b	10.41 @ 0.47ab	0.18 ± 0.00a
NSt-Sf	15.20 ± 0.60b	25.86 ± 1.52c	0.94 ± 0.01a	0.66 ± 0.02b	10.05 ± 0.72b	9.49 @ 0.74b	0.13 ± 0.00c
St-NSf	15.40 ± 0.50b	66.80 ± 3.78b	0.95 ± 0.03a	0.68 ± 0.05b	10.43 ± 0.36b	9.87 @ 0.62ab	0.15 ± 0.01b
St-Sf	27.05 ± 0.75a	75.13 ± 0.80a	0.80 ± 0.02b	0.56 ± 0.08c	15.27 ± 2.44a	12.20 @ 2.20a	0.08 ± 0.01d

The values are expressed as the mean ± standard deviation of triplicate experiments.

Values in the same column with different letters indicate significant differences (*P* < 0.05).

NSt-NSf, nonsteam and nonsulfur fumigation treatment; NSt-Sf, nonsteam and sulfur fumigation treatment; St-NSf, steam and nonsulfur fumigation treatment; St-Sf, steam and sulfur fumigation treatment.

The Sf treatment of *G. elata* also significantly increased the hardness and adhesiveness of starch gel. The hardness of the NSt-Sf and St-Sf treatments was 1.16 and 1.76-fold higher than that of the NSt-NSf and St-NSf treatments, respectively. The adhesiveness of the NSt-Sf and St-Sf treatments was 1.35 and 1.12-fold higher than that of the NSt-NSf and St-NSf treatments, respectively. Sf treatment decreased the cohesiveness and resilience of *G. elata* starch. The cohesiveness of the NSt-Sf and St-Sf treatments was 23 and 18% lower than that of the NSt-NSf and St-NSf treatments, respectively, and the resilience of the NSt-Sf and St-Sf treatments was 28 and 47% lower than that of the NSt-NSf and St-NSf treatments, respectively. Compared with the NSt-NSf, NSt-Sf, and St-NSf treatments, the St-Sf treatment decreased the springiness but significantly increased the gumminess and chewiness. The springiness decreased by 14, 15, and 16%, while the gumminess increased by 1.36, 1.52, and 1.46-fold, and the chewiness increased by 1.17, 1.29, and 1.24-fold, respectively (Table 2).

### 3.6. Effects of steaming and sulfur fumigation on FT-IR and XRD characteristics of *G. elata* starch

**FIGURE 5:**
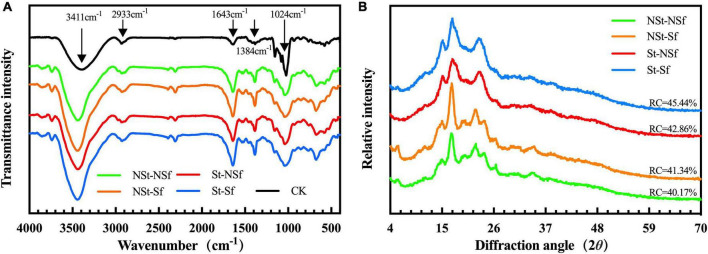
Fourier transform infrared spectroscopy (FT-IR) **(A)** and X-ray diffraction (XRD) **(B)** spectra of *Gastrodia elata* starch.

*Gastrodia elata* starch shows five absorption peaks under FT-IR: 3,411 cm^–1^ (O-H stretching), 2,933 cm^–1^ (C-H stretching), 1,643 cm^–1^ (C-O bending), 1,384 cm^–1^ (C-H bending), and 1,024 cm^–1^ (C-O stretching). A weak absorption peak was generated between 1,176 and 1,024 cm^–1^, which was caused by the tensile behavior of C-C and C-O. The weak absorption peak near 1,383 cm^–1^ was attributed to the bending vibration of -C-H, C-C-H, and C-O-H. Compared with CK (pure starch, V900508, Sigma-Aldrich Co., Ltd., Darmstadt, Germany), *G. elata* starch did not produce new absorption peaks after St treatment or Sf treatment, indicating that steaming or sulfur fumigation treatment did not alter the functional groups of *G. elata* starch (Figure 5A).

The XRD scanning results of *G. elata* starch showed diffraction peaks at 5.675°, 17.019°, 22.219°, and 24.031° after NSt-NSf and NSt-Sf treatments, indicating that the NSt treatment of *G. elata* starch was a typical B-type starch. Steamed *G. elata* lacked the diffraction peaks of St-NSf and St-Sf at 5.675°, and the double peaks of 22.219° and 24.031° merged into a single peak at 23.19°, indicating that the structure of the steamed *G. elata* starch changed to A-type (17). The relative crystallinity (RC) of starch treated with St-NSf and St-Sf increased by 1.17 and 2.58%, respectively, compared with NSt-NSf and NSt-Sf. Sulfur fumigation sharpened the characteristic peak shape of *G. elata* starch compared with that of nonfumigated starch, indicating enlarged grain size and increased RC. The RC of starch treated with NSt-Sf and St-Sf increased by 2.69 and 4.10%, respectively, compared with that of starch treated with NSt-NSf and St-NSf (Figure 5B).

### 3.7. Effect of steaming and sulfur fumigation on the RVA characteristics of *G. elata* starch

**FIGURE 6:**
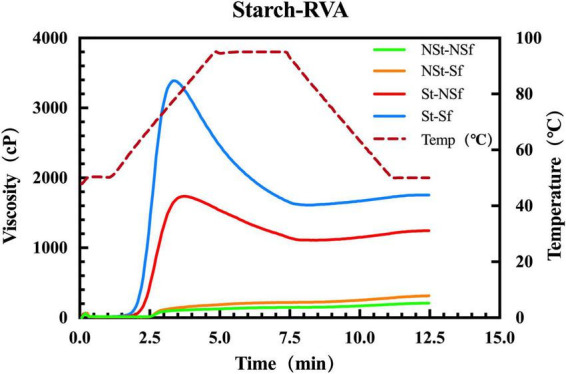
Rapid viscosity analyzer (RVA) of pasting properties of 5% *Gastrodia elata* starch.

During RVA, starches treated with St-NSf and St-Sf showed obvious peak viscosity (PV) and final viscosity (FV), but neither NSt-NSf nor NSt-Sf-treated starches reached PV and FV. Therefore, the PV and FV of St *G. elata* starch were further studied. After St treatment, the pasting temperature (Pt) of *G. elata* starch decreased, while PV and FV increased. Compared with *G. elata* starch treated with NSt-NSf and NSt-Sf, the Pt of the starch treated with St-NSf and St-Sf decreased by 11.7 and 15.47°C, respectively. The Sf treatment also reduced Pt and promoted the increase in PV and FV. Compared with starch treated with NSt-NSf and NSt-Sf, the Pt of starch treated with St-NSf and St-Sf decreased by 0.48 and 4.24°C, respectively, and the PV and FV of St-Sf increased by 1.95 and 1.33-fold, respectively, compared with St-NSf (Figure 6 and Supplementary Table 1).

### 3.8. Effect of steaming and sulfur fumigation on TG characteristics of *G. elata* starch

**FIGURE 7:**
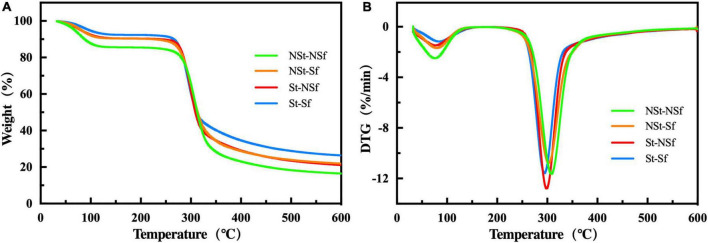
Thermogravimetric curves of weight changes with temperature **(A)** and derivative thermogravimetry (DTG) changes with temperature **(B)** of *Gastrodia elata* starch treated with nonsteam (NSt), steam (St) and nonsulfur fumigation (NSf), sulfur fumigation (Sf).

*Gastrodia elata* starch exhibits two rapid weight loss stages at 50–100°C and 250–350°C. The weight loss in the first stage is caused by the evaporation of crystal water in starch, while the second stage is attributed to the decomposition of starch. The presence of only two weight loss stages indicates that starch is a single component except water (Figures 7A, B). The St treatment decreased the degradation temperature (Td) of *G. elata* starch. The Td values of the St-NSf and St-Sf treatments were 8.6 and 8.5°C lower than those of the NSt-NSf and NSt-Sf treatments, respectively. The Sf treatment also decreased the Td value of *G. elata* starch. The NSt-Sf and St-Sf treatments decreased the Td values by 4.3 and 4.2°C, respectively, compared with the NSt-NSf and St-NSf treatments. St treatment reduced the loss of *G. elata* starch weight. The St-NSf and St-Sf treatments reduced the loss by 4.67 and 3.44%, respectively, compared with the NSt-NSf and NSt-Sf treatments. The Sf treatment also reduced the loss of *G. elata* starch weight. Compared with the NSt-NSf and St-NSf treatments, the weight loss of the NSt-Sf and St-Sf treatments decreased by 6.49 and 5.26%, respectively (Supplementary Table 1).

### 3.9. Effects of steaming and sulfur fumigation on rheological characteristics of *G. elata* starch

**FIGURE 8:**
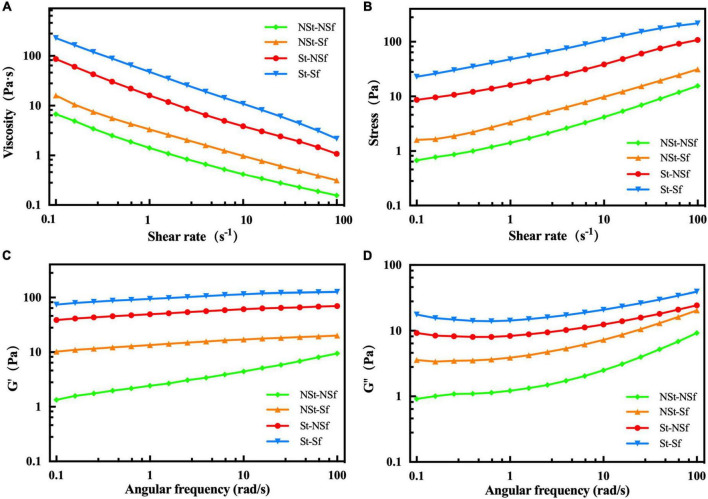
Stable shear flow curves of viscosity changes with shear rate **(A)** and stress changes with shear rate **(B)**. The changes in storage modulus (G′) **(C)** and loss modulus (G′′) **(D)** of *Gastrodia elata* starch at 25°C and 1% strain.

The apparent viscosity of *G. elata* starch decreased with increasing shear rate, showing the characteristics of pseudoplastic fluid (Figure 8A). St-NSf and St-Sf treatment led to a significant increase in the apparent viscosity of starch compared with that of NSt-NSf and NSt-Sf treatment. In addition, the NSt-Sf and St-Sf treatments also increased the apparent viscosity of starch compared with the NSt-NSf and St-NSf treatments. The rheological behavior of starch shown in Figure 8B was fitted with a power law equation, and the results showed that all non-Newtonian indices (n) were less than 1, indicating that the samples were all non-Newtonian fluids. The *n* values of the St-NSf and St-Sf treatments were significantly higher than those of the NSt-NSf and NSt-Sf treatments, indicating that steaming enhanced the pseudoplasticity of the starch gel, and sulfur fumigation also enhanced the pseudoplasticity of the starch gel (Supplementary Table 2). In addition, the increased value of the consistency coefficient K in the fitted equation also indicates that both steaming and sulfur fumigation can increase the viscosity of *G. elata* starch.

The storage modulus (G′) and loss modulus (G′′) gradually increase with increasing angular frequency, and G′ > G′′, indicating that *G. elata* starch is a weak gel (Figures 8C, D). The G′ and G′′ of steaming treatments (St-NSf and St-Sf) were significantly higher than those of nonsteaming treatments (NSt-NSf and NSt-Sf), indicating that the degree of molecular crosslinking of gelatinized *G. elata* starch solution was significantly enhanced by the St treatment. The G′ and G′′ of the sulfur fumigation treatment (NSt-Sf and St-Sf) were significantly higher than those of the nonfumigation treatment (NSt-NSf and St-NSf), indicating that the degree of molecular crosslinking of gelatinized *G. elata* starch solution was also significantly enhanced following sulfur fumigation.

### 3.10. Effect of steaming and sulfur fumigation on the molecular weight distribution of *G. elata* starch

**FIGURE 9:**
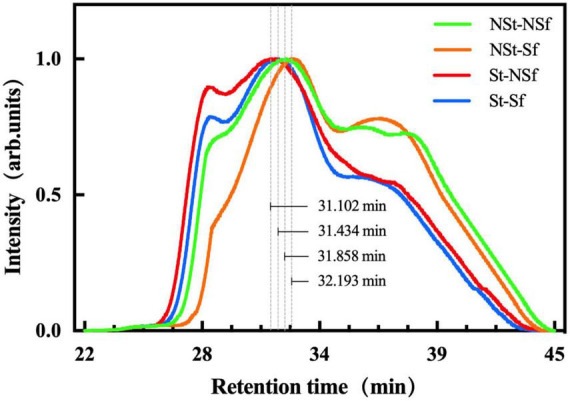
Molecular weight and size distribution of *Gastrodia elata* starch (with normalized intensity).

The high-molecular-weight amylopectin in *G. elata* starch eluted at 22–34 min, and the low-molecular-weight amylose eluted at 34–45 min (Figure 9). After normalizing the intensity, the peak time of *G. elata* amylopectin elution after steaming was earlier than that of nonsteamed amylopectin. St-NSf and St-Sf were 0.76 and 0.76 min earlier than NSt-NSf and NSt-Sf; the number-average molecular mass (Mn) was 1.27 and 1.49-fold higher; the weight-average molecular mass (Mw) was 1.30 and 1.53-fold higher; and the polydispersity index (PDI) was 1.03 and 1.02-fold higher, respectively. After Sf treatment, the peak time of *G. elata* amylopectin elution was earlier than that of NSf-treated amylopectin. Compared with NSt-NSf and St-NSf, NSt-Sf and St-Sf increased by 0.34 and 0.33 min; Mn decreased by 17.55 and 2.94%; Mw decreased by 18.34 and 4.14%; and PDI decreased by 1.09 and 1.60%, respectively (Supplementary Table 3).

### 3.11. Effect of steaming and sulfur fumigation on the *in vitro* digestibility of *G. elata* starch

**FIGURE 10:**
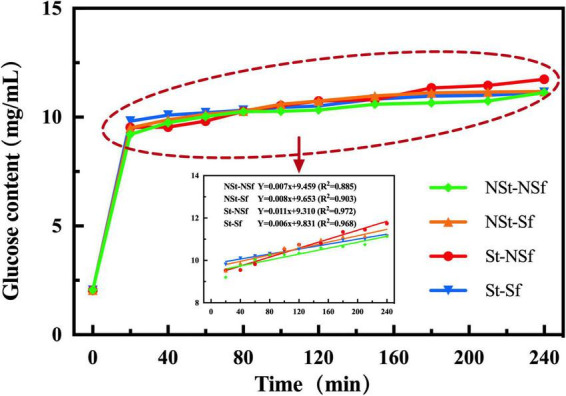
Digestion of *Gastrodia elata* starch in 4 h.

The RSD content of *G. elata* was significantly increased after steaming. Compared with NSt-NSf and NSt-Sf, it was 1.05 and 1.04-fold higher under St-NSf and St-Sf treatments, respectively. Sulfur fumigation also promoted an increase in the RSD content of *G. elata*. Compared with the NSt-NSf and St-NSf treatments, the RSD content under NSt-Sf and St-Sf increased by 1.04 and 1.04-fold, respectively. However, the effect of steaming and sulfur fumigation on the levels of SDS and RS showed no obvious change (Table 3). The linear fitting results indicated that the starch digestion rate from 20 to 240 min under the four treatments was significantly higher under steaming than under nonsteaming conditions, indicating the enhanced stability of the starch hydrolysis rate after steaming treatment (Figure 10).

**TABLE 3:** Effects of steaming and sulfur fumigation treatment on different starch contents in *Gastrodia elata.*

Sample	RSD (%)	SDS (%)	RS (%)
NSt-NSf	80.34 ± 0.08c	12.73 ± 0.08b	6.93 ± 0.00a
NSt-Sf	83.85 ± 0.19b	13.93 ± 0.27a	2.22 ± 0.08c
St-NSf	84.14 ± 0.00b	13.61 ± 0.04a	2.25 ± 0.04c
St-Sf	87.35 ± 0.08a	7.97 ± 0.00c	4.68 ± 0.08b

The values are expressed as the mean ± standard deviation of triplicate experiments.

Values in the same column with different letters indicate significant differences (*P* < 0.05).

RSD, rapidly digesting starch; SDS, slowly digest starch; RS, resistant starch.

NSt-NSf, nonsteam and nonsulfur fumigation treatment; NSt-Sf, nonsteam and sulfur fumigation treatment; St-NSf, steam and nonsulfur fumigation treatment; St-Sf, steam and sulfur fumigation treatment.

### 3.12. Effect of steaming and sulfur fumigation on the morphology of *G. elata* starch granules

**FIGURE 11:**
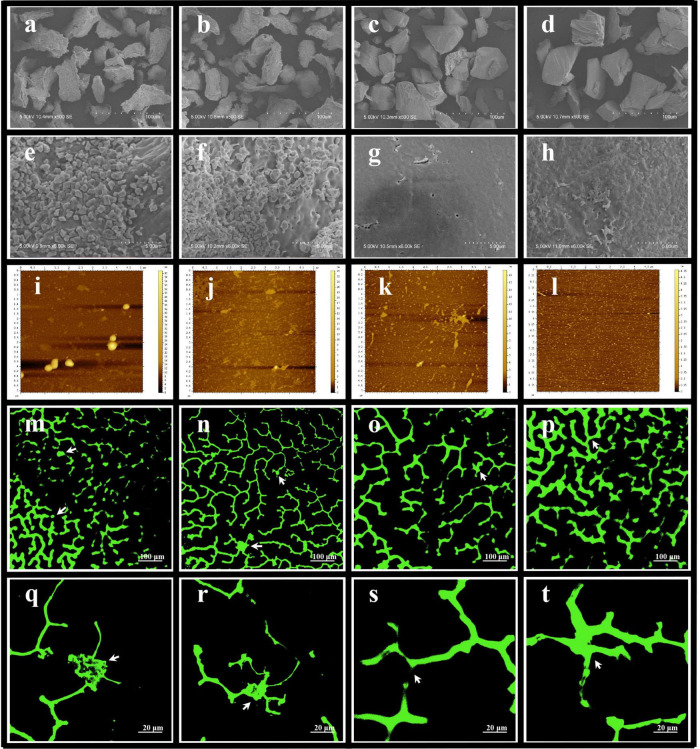
Effect of steaming and sulfur fumigation on the morphology of *Gastrodia elata* starch granules. Scanning electron microscopy (SEM) images of *G. elata* starch granules [**(a,e)** nonsteam and nonsulfur fumigation treatment (NSt-NSf); **(b,f)** nonsteam and sulfur fumigation treatment (NSt-Sf); **(c,g)** steam and nonsulfur fumigation treatment (St-NSf); **(d,h)** steam and sulfur fumigation treatment (St-Sf)]. AFM images of *G. elata* starch granules after gelatinization for 10 min of NSt-NSf **(i)**, NSt-Sf **(j)**, St-NSf **(k)**, St-Sf **(l)**. Confocal experimental images of *G. elata* starch granules after gelatinization for 10 min [**(m,q)** NSt-NSf; **(n,r)** NSt-Sf; **(o,s)** St-NSf; **(p,t)** St-Sf].

The starch granuless of nonsteamed *G. elata* (NSt-NSf and NSt-Sf) was finer and irregular (Figures 11A, B, E, F). Steaming resulted in fusion of the small granules on the surface of the starch granules, resulting in a flat and smooth texture (Figures 11C, D, G, H). Compared with the St-NSf treatment, sulfur fumigation (St-Sf) roughens the surface of the *G. elata* starch granules, and the structures swell and are even destroyed (Figures 11C, D). Sulfur fumigation (NSt-Sf) decreased the diameter of the starch granules from 0.5 to 0.1–0.3 μm and changed the irregular shape to a near circular shape (Figures 11E, F). Sulfur fumigation (St-Sf) also increased the surface roughness of the steamed starch granules (Figures 11G, H).

Compared with NSt treatments (Figures 11I, J), the gelatinized starch diluent of *G. elata* was reduced in height and decreased in size in the St treatments (Figures 11K, L), indicating increased leaching of starch chains from the starch granules. Compared with the NSf treatment (Figures 11I, K), the Sf treatment (Figures 11J, L) decreased the granule height and significantly reduced the large starch granules, indicating that sulfur fumigation also promotes the leaching of starch chains from the granules. Among the four treatment conditions, the *G. elata* starch granules treated with St-Sf showed the least height and exhibited uniform granule shape and size, which indicates that sulfur fumigation after steaming was the most appropriate strategy to promote the leach out of starch chains from starch granules.

Figures 11M–T green fluorescence shows the reticular structure formed by cross-linking of leached out starch chains from starch granules after starch gelatinization. In Figure 11M, the light and dark distribution of green fluorescence without steaming and sulfur fumigation (NSt-NSf) is uneven, and there is an obvious granular structure (as indicated by the white arrow in Figure 11M), which shows that starch granules without steaming and sulfur fumigation treatment are not completely leached out during gelatinization. Compared with the treatment of NSt-NSf and St-NSf (Figures 11M, O, Q, S), the reticular structure (Figures 11N, P, R, T) after treatment of NSt-NSf and St-Sf is clearer and more evenly distributed, which indicates that sulfur fumigation can promote the leaching of starch chains from starch granules. Compared with the nonsteaming treatment (NSt-NSf and NSt-Sf, Figures 11M, N), the steaming treatment (St-NSf and St-Sf) has a more complicated degree of interlacing and winding of the reticular structure formed by the starch chains leached out from the starch granules (Figures 11O, P), which is due to the traction effect of forming hydrogen bonds between short amylose chains produced by steaming with long starch chains. In addition, compared with that without steaming (Figures 11Q, R), no obvious starch granules are found after the starch granules treated by steaming are gelatinized (Figures 11S, T), which indicates that starch chains are almost completely leached out from starch granules.

## 4. Discussion

After washing the fresh *G. elata*, it was steamed under normal pressure for 30 min until no white core was observed in the cross section. Sulfur and *G. elata* were placed in an enclosed fumigation chamber based on specific proportions and ignited the sulfur, which is the traditional processing technology of *G. elata* (25). The processing technology of *G. elata* has an 1,800-year history. Although it has been increased several times, the basic operating process of steaming and sulfur fumigation has not changed, suggesting that these two methods play a decisive role in the quality of *G. elata*. However, their effects on the taste, perception and quality of *G. elata* and the mechanism of action still lack scientific research. To validate the effect of steaming and sulfur fumigation on *G. elata* taste, sensation and quality during manufacture, this study focused on the changes in starch properties that are most closely related to the quality of *G. elata* and show the highest content.

### 4.1. Steaming and sulfur fumigation changed the morphology of *G. elata* starch

The morphological quality of starch is mainly measured in terms of color, which is usually described qualitatively based on whiteness, brightness and paste transparency. Light, bright, and transparent starch is more popular with consumers. In this study, both steaming and sulfur fumigation significantly increased the whiteness, brightness and paste transparency of *G. elata* starch, indicating that the traditional processing technology of *G. elata* can increase the starch morphology.

Polyphenols in fresh fruits and vegetables will enzymatically brown under the action of polyphenol oxidase to produce brown substances, which will reduce their morphology quality. In the food industry, the color quality of fruits and vegetables is often increased by inhibiting the activity of browning enzymes (26). For example, Hemachandran et al. (27) used cyanidin-3-sophoroside in mangosteen peel to reduce the activity of polyphenol oxidase in a noncompetitive manner, thereby inhibiting the browning of apple and increasing its antioxidant ability. In this study, the whiteness of steamed *G. elata* starch was significantly higher than that of nonsteamed starch because high-temperature steaming inactivated the browning enzyme in fresh *G. elata* and reduce enzymatic browning. Sulfur fumigation generates SO_2_ after combustion, and the gas combines with water in the fumigated product to form sulfite (15). Sulfite is a weak acid that reduces the pH of fumigated substances. Liu et al. (19) reported that after fumigating ginseng with sulfur, the pH of ginseng decoction decreased from 5.32 to 5.09. In this study, the pH of the extract of sulfur-fumigated *G. elata* was significantly lower than that of the nonfumigated starch, which indicates that the sulfur-fumigated *G. elata* exhibited the effects of fumigation in this study. Sulfur fumigation is based on the principle of sulfite generation and reaction with organic pigments to form colorless compounds, which inhibit nonenzymatic browning to achieve the bleaching effect (28). In addition, sulfites also form salts to induce antiseptic and antioxidant effects. In this study, the significant increase in the whiteness of *G. elata* after sulfur fumigation was attributed to the bleaching effect of sulfurous acid generated after fumigation on organic pigments and the inhibition of enzymes with a browning effect, as well as the reduction in the accumulation of pigments produced during microbial reproduction. The combined action increases the whiteness (28, 29).

Nonetheless, we also observed a very interesting phenomenon. Without sulfur fumigation, the pH of *G. elata* after St treatment was significantly higher than the pH of NSt treatment. Jorge et al. (30) and Jorge et al. (31) reported that cooking and steaming resulted in damage to the plasma membrane of cabbage and increased permeability. Organic acids such as oxalic acid, citric acid, and ascorbic acid spill out of the substances during high-temperature cooking, and acidic substances are lost. Compared with fresh cabbage, the pH increased by approximately 0.15. Therefore, we speculate that the increase in the pH of *G. elata* after steaming may be related to the loss of organic acids. However, irrespective of *G. elata* steaming, no significant difference in pH was observed after sulfur fumigation. This is because the pH decrease (0.977) induced by sulfur fumigation is significantly higher than the pH increase (0.578) due to steaming, resulting in a relatively small effect of steaming on pH.

Paste transparency is affected by the color of starch and the size of granules in the solution. The lighter the color and smaller the starch granules are, the higher the paste transparency (32). To increase the quality of starch, starch modification is often used to increase its paste transparency. For example, Lee et al. (11) reported that the paste transparency of pregelatinized rice starch after dry heat treatment at 130°C for 3 h was 1.02-fold higher than that in the absence of dry heat treatment. In addition, Ali et al. (12) reported that acetylation of starch with acetic acid was also effective in increasing the transparency of starch paste. In this study, the increase in the transparency of starch paste was attributed to the increased whiteness of *G. elata* starch promoted by steaming and sulfur fumigation. However, steaming and sulfur fumigation decreased the granule size of gelatinized starch. Compared with sulfur fumigation, steaming has a more significant effect on increasing the transparency of starch paste. However, sulfur fumigation can further consolidate and enhance the effect of steaming on the transparency of starch paste. It can be seen that the combination of steaming and sulfur fumigation can enhance the whiteness and paste transparency of *G. elata* starch.

### 4.2. Steaming and sulfur fumigation increase the pasting properties and chewiness of *G. elata* starch

The taste index of starch usually refers to the hardness, chewiness and viscosity of the gel. An appropriate gel hardness and viscosity will soften the food but will not be loose and soft or sticky when eating. If the index is very low, it will be loose and tasteless. If it is excessively high, it will be hard to chew. In this study, the branched chains of *G. elata* starch were broken and polymerized after sulfur fumigation, resulting in an increase in final viscosity (FV) and chewability. This is consistent with the research conclusion of Xie et al. (33).

**FIGURE 12:**
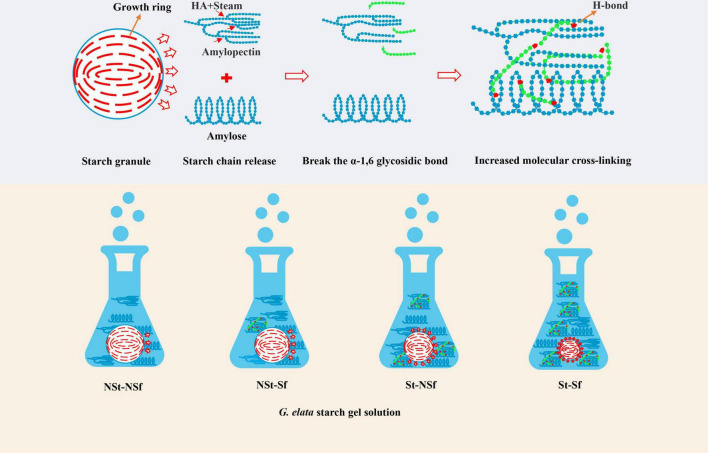
Schematic diagram of starch cross-linking degree change of *Gastrodia elata* after steaming and sulfur fumigation process.

As cooking indicators, solubility and swelling power refer to the mass loss of dry matter and the change in water absorption of food after cooking. Hormdok et al. (34) reported that low solubility and limited swelling can stabilize the structure of starch gel, which can be described as low solubility and limited swelling and water absorption to enhance the food taste by cooking. Li et al. (35) showed that the cooking quality of noodles with high solubility is worse, and a high degree of swelling results in a soft texture of the noodles, while an extremely low degree of swelling results in a hard texture. Lu et al. (20) also showed that the increased degree of swelling in a solution with a specific water content promotes cross-linking between starch chains and thus enhances the gel strength. The amylose content alters the degree of dissolution and swelling. The results of Yang et al. (36) show that starch with high amylose content is more difficult to dissolve. In addition, Singh et al. (37) reported that the swelling degree of wheat starch was negatively correlated with the amylose content (*r* = −0.444, *P* = 0.06). Amylopectin α-1,6 glycosidic bonds form amylose after hydrolysis and debranching, and the unbranched chains become short amylose chains (38). The results of Yang et al. (39) show that the corn starch α- 1,4 glycoside bond is more stable than the α- 1,6 glycoside bond, which indicates that under acidic conditions, α- 1,6 glycosidic bonds are preferentially hydrolyzed. Gunaratne et al. (13) treated wheat, potato, and corn starch with 0.1 mol/L dilute hydrochloric acid for 1.5 h, which increased the amylose content by 1.07, 1.04, and 1.07-fold, respectively. Chen et al. (15) also reported that the contents of amylose and total starch in yam after sulfur fumigation increased by 1.05 and 1.04-fold compared with those of the nonsulfur fumigation treatment, respectively. In this study, the amylose content after St-Sf treatment was 1.10-fold higher than that of NSt-NSf treatment. This is because, on the one hand, the high-temperature treatment induces depolymerization and molecular degradation of starch granules and increases the amylose content (16). On the other hand, the weak acidic conditions formed by sulfite during sulfur fumigation of *G. elata* also promote the preferential hydrolysis of more unstable α- 1,6 glycosidic bonds of *G. elata* amylopectin to form short amylose chains and increase their content (15, 39). In this study, the elution peak time of amylopectin after sulfur fumigation is longer than in the absence of sulfur fumigation. This indicates that amylopectin is degraded, which proves that the sulfite we mentioned earlier can hydrolyze α- 1,6 glycosidic bonds, speculation of increasing amylose content. However, the elution peak time of amylopectin after steaming treatment is advanced, and Mn and Mw are significantly increased, which may be caused by the aggregation of hydrogen bonds between starch chains, the molecules formed by this aggregation are not easy to separate in the chromatographic column, thus showing higher molecular weight (Figure 12; 40). Interestingly, the Mn and Mw of starch molecules after steaming are not significantly affected by sulfur fumigation, which may be attributed to the breakage of most molecular chains caused by steaming, so that the sulfites formed by sulfur fumigation cannot break additional molecular chains. To summarize, it can be seen that steaming and sulfur fumigation in this study not only reduce the solubility and increase the swelling degree of starch by changing the molecular crosslinking degree of starch but also further reduce the solubility of starch and limit the excessive increase in swelling degree by increasing the content of amylose, ultimately promoting the increase in starch gel strength.

During cooking, with the gelatinization of starch and the destruction of crystal structure, amylose, and amylopectin chains are leached from starch granules, and the branched structure of the chain is connected by hydrogen bonds, resulting in stronger viscosity (9). Although the structure of starch chains cannot be observed at the granule level, it can be inferred from the height of starch granules. The lower the height of starch granules, the higher the degree of starch chains leached out from starch granules (41). In this study, the AFM results showed that sulfur fumigation and steaming reduced the height of starch granules in starch gelatinization solution, which proved that the leaching out of starch chains was significantly increased. With the gradual increase in the leach out of starch chains from the granules, the crosslinking of the starch chains was enhanced, thus strengthening the gel structure of starch. This is the reason for the increase in starch gel hardness and viscosity, PV and FV during RVA, G′ and G′′ modulus in rheology. Finally, the chewability and sticky taste of *G. elata* starch gel was increased.

### 4.3. Steaming and sulfur fumigation change the structure and digestibility of *G. elata* starch

Starch is the main energy source of human beings. The digestible RSD content is an important index of food quality. The high temperature destroys the double helical structure of starch molecules, reduces the crystal stability and generates further short-chained molecules, resulting in increased RSD content (42). In addition, the release of starch chains caused by high temperature is completely exposed to the sulfurous acid environment produced by sulfur fumigation, which aggravates the breaking of α- 1,6 glycosidic bonds (15, 39, 41). In this study, the heat loss rate of starch after steaming treatment and sulfur fumigation treatment was reduced, and the relative crystallinity was increased. Because it promotes the leaching out and exposure of starch chains from granules, it not only forms more short starch chains but also reduces the size of starch granules, increasing their contact area with hydrolytic enzymes and leading to a significant increase in starch RSD content.

Fourier transform infrared spectroscopy (FT-IR) and XRD can reflect the molecular structure and crystal structure of starch. In this study, there was no significant difference between the functional groups of standard starch (CK) and *G. elata* starch under the four processing methods (Figure 5A), which indicates that the extracted *G. elata* starch does not contain protein. TG test results reveal no other loss except for the loss of significant mass in the two stages caused by crystal water and starch molecule loss. Thus, the presence of no other substances except starch in the extracted *G. elata* starch indicates the absence of impurities affecting the structure and pasting performance of starch. The XRD results showed that steaming induced the transformation of *G. elata* starch crystals from a B-type structure to an A-type structure and increased the relative crystallinity of starch. This can be attributed to the opening of the B-type structure of starch caused by steaming, and the water molecules enter the double helix channel. Then, loss of water leads to rearrangement of the crystal structure into a more stable A-type crystal (17). Moreover, the movement of water molecules inside starch induced by high-temperature treatment of starch alters the direction of the starch double helix in the crystal region, resulting in a highly ordered crystal configuration and thereby increasing the relative crystallinity of starch (17). Sulfur fumigation has no significant effect on the crystal structure of *G. elata* starch, but it increases the relative crystallinity, which may be due to the possible hydrolysis of the noncrystalline region of starch under the weakly acidic environment induced by sulfur fumigation, followed by the crystalline region (17). In addition, the digestibility of starch is also related to its crystal structure. According to Xie et al. (43), type A starch is easier to digest than type B starch. In this study, steaming changed the crystalline form of *G. elata* from type B to type A, accompanied by an increase in RSD content. Therefore, we speculate that the altered crystal structure of *G. elata* starch is also one of the factors contributing to the increased RSD content.

## 5. Conclusion

This study showed that sulfur fumigation after steaming could not only increase the morphological quality of *G. elata* starch by increasing the whiteness and paste transparency through the bleachability of sulfite but also cause the degradation of amylopectin to produce amylose and promote an increase in RSD content to increase its edible quality. In addition, the release degree of starch chains from starch granules was increased by sulfur fumigation after steaming, thus enhancing the pasting characteristics of gel and cooking qualities such as hardness, chewiness, and stickiness, which meant that the taste quality of starch was increased. This work explained the improvement mechanism of steaming and sulfur fumigation treatment on *G. elata* edible quality and provided a scientific basis for the rational use of sulfur fumigation technology in food processing. This modified starch method may be used to improve the gel taste of low viscosity food and the digestion rate of starch products.

## Data availability statement

The original contributions presented in this study are included in the article/Supplementary material, further inquiries can be directed to the corresponding authors.

## Author contributions

JG, ZC, and YY wrote and reviewed the manuscript. TZ, JG, TX, and FW collected the samples. JG, ZC, LG, CW, and YY provided analysis ideas and reviewed the manuscript. JG and ZC reviewed the manuscript and drew the picture. LG and XC supervised the work and read the manuscript. All authors contributed to the article and approved the submitted version.
