# Supplementary material for: Evaluate how steaming and sulfur fumigation change the microstructure, physicochemical properties and in vitro digestibility of Gastrodia elata Bl. starch
Source: Front Nutr. 2023 Jan 5;9:1087453. doi: 10.3389/fnut.2022.1087453 (PMC9849879; doi:10.3389/fnut.2022.1087453)
Supplement: Supplementary file 1 [file Table_1.DOCX]

**Supplementary material:**

Table S1 Pasting and thermogravimetric properties of starch

| Sample | Pasting properties | | | Thermal properties | |
| --- | --- | --- | --- | --- | --- |
|  | Pt（℃） | PV（cP） | FV（cP） | Td（℃） | Weight loss（%） |
| NSt-NSf | 67.68±0.67a | 336.00±31.11c | 336.00±31.11c | 308.20±0.40a | 83.52±0.11a |
| NSt-Sf | 67.20±0.00a | 313.50±14.85c | 313.50±14.85c | 303.90±0.71b | 77.03±1.00b |
| St-NSf | 55.97±1.94b | 1743.00±374.41b | 1323.67±54.31b | 299.60±0.30c | 78.85±0.70c |
| St-Sf | 51.73±3.02b | 3399.50±504.48a | 1754.25±87.21a | 295.40±0.57d | 73.59±0.38d |

The values were expressed as the mean ± standard deviation of triplicate experiments.

Values in same column with different letter indicated significant difference (P <0.05).

Pt, Pasting temperature. PV, Peak viscosity. FV, Final viscosity. Td, Degradation temperature.

Table S2 Flow fitting parameters of *G*.*elata* starch

| Sample | K | n | R^2^ |
| --- | --- | --- | --- |
| NSt-NSf | 1.22d | 0.55a | 0.998 |
| NSt-Sf | 3.21c | 0.49b | 0.999 |
| St-NSf | 15.36b | 0.43c | 0.996 |
| St-Sf | 50.14a | 0.33d | 0.995 |

The values were expressed as the mean ± standard deviation of triplicate experiments.

Values in same column with different letter indicated significant difference (P <0.05).

K, Consistency coefficient (Pa·s). n, non-newtonian index. R^2^, Fitting coefficient.

Table S3 Molecular weight distribution of *G.elata* starch after steaming and sulfur fumigation treatment

| Sample | Mn×10^5^（g/mol） | Mw×10^5^（g/mol） | PDI |
| --- | --- | --- | --- |
| NSt-NSf | 3.76±0.14b | 6.87±0.09b | 1.83±0.05b |
| NSt-Sf | 3.10±0.09c | 5.61±0.07c | 1.81±0.01b |
| St-NSf | 4.76±0.11a | 8.94±0.13a | 1.88±0.04a |
| St-Sf | 4.62±0.08a | 8.57±1.14a | 1.85±0.02b |

The values were expressed as the mean ± standard deviation of triplicate experiments.

Values in same column with different letter indicated significant difference (P <0.05).

Mn, number-average molar mass. Mw, weight-average molar mass. PDI, polydispersity index (PDI) = Mw/Mn.
